# Incidence of surgical site infections in cerebrovascular surgery: a single-center cohort study

**DOI:** 10.1016/j.bas.2025.104389

**Published:** 2025-08-06

**Authors:** Elisa Colombo, Alexandra Grob, Johannes Sarnthein, Jorn Fierstra, Giuseppe Esposito, Luca Regli, Menno Germans

**Affiliations:** Department of Neurosurgery and Clinical Neuroscience Center, University Hospital of Zurich, University of Zurich, Switzerland

**Keywords:** Complication, Surgical site infections, Cerebrovascular, Craniotomy

## Abstract

**Introduction:**

Surgical site infections (SSI) constitute a source of morbidity in neurosurgical patients. Few reports address incidence of SSIs in patients undergoing craniotomy for neurovascular diseases.

**Research question:**

This study aims to determine incidence and characteristics of SSI with interval to diagnosis, severity, and type of pathogen after intracranial neurovascular surgery.

**Materials and methods:**

Data from consecutive patients undergoing intracranial neurovascular surgery between January 2013 and 2022 were retrospectively analyzed in our tertiary referral center. Demographic data, risk factors, treatment and hospital stay were collected. Impact of SSI were classified according to Clavien Dindo Grade (CDG). Parameters studied included the time from index to revision surgery, microorganisms involved, and underlying pathology for the index surgery.

**Results:**

Among 1065 neurovascular surgeries, SSI occurred in 34 cases (2.1 %, n = 19 males). Median patient's age was 53 years (interquartile range, IQR 45–60). The median time from the index procedure to surgical wound revision was 27.5 days (IQR 19–44). The median length of hospital stay after revision surgery was 7 days (IQR 4–15). The predominant pathogen was Staphylococcus epidermidis (11, 32 %), followed by Staphylococcus aureus (8, 22 %). In 32 cases (89 %) wound revision was performed under general anesthesia.

**Discussion and conclusions:**

The overall incidence of SSI in our center was 2.1 % with a mean time to wound revision of 29 days. These numbers can be used as a benchmark and reference for strategies that implement a reduction of postoperative infections in neurovascular surgery.

## Highlights

• Retrospective analysis from 1065 prospective neurovascular surgeries over nine years in a tertiary referral center.
• SSI incidence of 2.1 %, with a mean interval to wound revision of 34 days.
• Predominant *Staphylococcus epidermidis* and *Staphylococcus aureus*, impacts classified using the Clavien-Dindo Grading.
• These results provide valuable insights and a benchmark for preventive strategies in cerebrovascular surgery.
• This work fills a gap in the literature by presenting detailed data on SSI in intracranial neurovascular procedures.

## 1 Introduction

Surgical site infections (SSI) represent a significant burden for the healthcare system worldwide, complicating many surgical procedures and increasing postoperative morbidity and mortality (Anderson and Kaye, 2009; Korol et al., 2013). Severity of SSIs varies according to the depth of the infection, the microbiological agent involved and the baseline conditions of the patients and can range from superficial infections to life-threatening conditions. Regardless of the severity of SSIs, the impact of these healthcare-associated infections is inevitably negative for the general physical and psychological well-being of patients (Korol et al., 2013).

Previous reports have documented an overall incidence of SSIs averaging 5 % for general surgery (Anderson and Kaye, 2009; Zarb et al., 2012; Borchardt and Tzizik, 2018). According to the published literature, the incidence of SSIs after intracranial neurosurgical procedures overall, i.e. including traumatic and non-traumatic indications, ranges between 1.2 and 5.5 % (Magni et al., 2024; Lee et al., 2024). Few studies also attempted the identification of risk factors with the goal to develop strategies to prevent postoperative SSIs by implementing preventive perioperative strategies (McClelland, 2008; Cassir et al., 2015; Stienen et al., 2019; Lee et al., 2024). Intracranial pathologies regularly require complex surgical procedures of long duration, and among them neurovascular lesions represent a specific category in terms of surgical complexity (Dammann et al., 2014).

Intracranial vascular surgery is widely recognized as one of the most complex areas within neurosurgery (Gross and Sacho, 2021). This complexity is largely due to the delicate nature of the pathologies being treated and their location within densely packed, highly sensitive regions of the brain. Surgeons must navigate tight anatomical spaces, often working around critical vessels and neural structures where even minor missteps can have serious consequences. The technical precision required, combined with the fragility of the tissues affected, makes these procedures particularly demanding and leaves little room for error. This challenging environment can influence not only the surgical approach but also the risks and outcomes, including the potential for complications such as surgical site infections. Nonetheless, a prospective study reporting SSI specific to intracranial neurovascular surgery is still lacking.

We present a detailed analysis of a single-center cohort of patients who underwent surgery for an intracranial vascular pathology and developed a postoperative SSI. The aim of this study is to provide a benchmark for further defining the incidence of surgical site infections in cerebrovascular surgery.

## 2 Materials and Methods

Patient data and outcomes were prospectively collected in an institutional patient registry, which was approved by the local ethics review board (Kantonale Ethikkommission, PB-2017-00093) and internationally registered at clinicaltrials.gov (identifier no. NCT01628406).

### 2.1 Patient cohort

We included all consecutive patients that underwent craniotomy for cerebrovascular surgery in our Institution between January 2013 and January 2022. Our institution represents a tertiary referral center for the management of complex cerebrovascular pathologies, with a volume of more than 200 craniotomies per year and an interdisciplinary team for the most optimal and up-to-date evaluation of these diseases.

Specific inclusion criteria were craniotomy and/or craniectomy performed in elective, semi-elective and emergency settings for cerebrovascular surgical cases. Cerebrovascular surgery included pathologies that involve the major vascular supply of the brain, such as ruptured and unruptured intracranial aneurysms, extracranial-intracranial bypasses and vessel malformations (Cerebrovascular Surgery). All patients must have been above 18 years old and have consented for the anonymized use of their demographic, medical and radiological data for research purposes to be included in the study. Hemicraniectomies for large strokes were included when the surgical procedure represented the primary therapy for the direct consequences of the stroke, such as malignant edema. We excluded carotid endarterectomies (CEAs) and neurosurgical procedures associated with traumatic brain injury (e.g., post-traumatic acute or chronic subdural hematoma, intraparenchymal bleeding, and traumatic subarachnoid hemorrhage) as well as spontaneous hypertensive brain hemorrhages and hemorrhages caused by microangiopathic changes (i.e. cerebral amyloid angiopathy).

We collected demographics, indications for and location of index surgery, length of the initial hospital stay and interval to diagnosis of infection. Additionally, functional outcome was measured, assessing the means of modified Rankin Scale (mRS) and National Institute of Health Stroke Scale (NIHSS) at the time of hospital admission and discharge for the infection. Perioperative treatment with corticosteroids and information regarding an existing immunosuppression prior to initial surgery were collected.

### 2.2 Perioperative standard operation protocol

Our perioperative standardized management was performed in all surgeries. All index surgeries were performed under general anesthesia. Antibiotic prophylaxis (Cefuroxim, a 2nd generation cephalosporin weight-adjusted, and if necessary, repeated every 4 h) was administered within 60 min before skin incision. A localized area of the scalp along the incision, ca 2 cm in width, was shaved in all cases prior to disinfection. Wound preparation before skin incision was performed for each craniotomy with the broad-spectrum antiseptic Chlorhexidine 2 %, which combines 2 % chlorhexidine gluconate and 70 % isopropyl alcohol. A layer-by-layer wound closure was performed in all surgical cases using 2-0 braided resorbable inverted sutures for the subcutaneous layer and surgical staples for the cutaneous layer in patients operated for the first time, except in the posterior fossa. Alternatively, in patients undergoing recurrent operations and for all surgeries on the posterior fossa, 2-0 monofilament non-resorbable simple or continuous (occipital wounds) sutures were used to close the cutaneous layer. Sterile wound covering postoperatively and daily wound checks starting after the second postoperative day were performed routinely. Suture material was removed between day 8 and day 14, depending on the localization of the wound and neurosurgeon's preference.

### 2.3 Assessment of surgical site infections

**Fig. 1:**
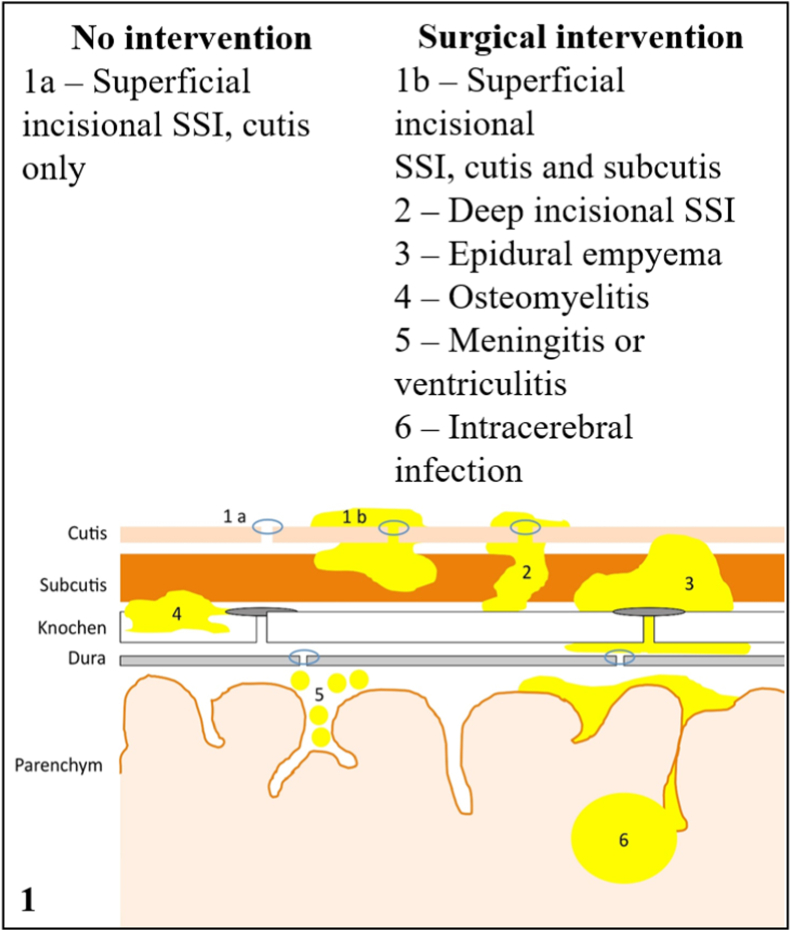
Schematization of the classification of SSIs according to the depth and the degree of tissue involvement.

**Fig. 2:**
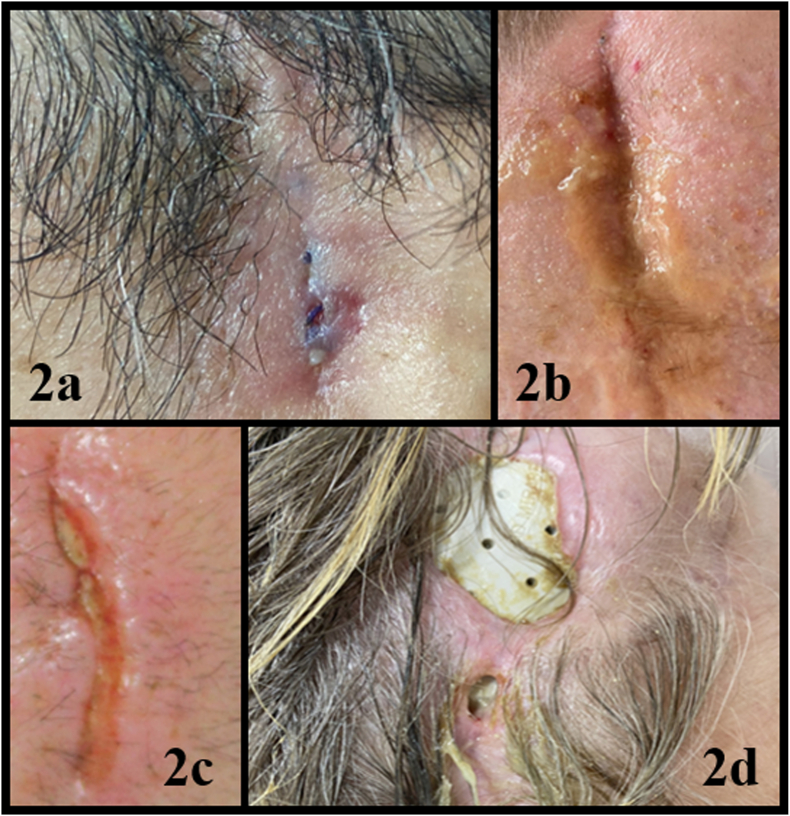
Different presentations of SSI at the time of diagnosis. According to the schematization presented in Fig. 1 and used in our center: SSI type 1b is shown in 2a and 2b. SSI type 2 is demonstrated in 2c and finally SSI type 3 with skin erosion and visualization of a cranioplasty is shown in 2b.

SSI were defined according to the *Centers for Disease Control and Prevention* criteria and treated according to our local standardized protocol for SSI (Fig. 1, Fig. 2). SSI were diagnosed by means of inspection of the wound, bloodwork aiming to study parameters showing infections, performance of contrasted imaging to identify the depth of the infection prior to the eventual surgical treatment and, indeed, a microbiological analysis was performed for all patients to identify the causative microorganism. Severity of SSI was rated according to the established Clavien Dindo Grading (CDG), which indicated type of therapeutic intervention required after diagnosis of a postoperative adverse event as visually shown in Table 1 (Clavien et al., 2009; Dindo et al., 2004). Type of surgical revision for SSI, intraoperative microbiological probes from different tissues (dura, bone, subcutaneous and if possible, implants) and antibiotic treatment course were analyzed.

**Table 1:** Clavien-Dindo classification of surgical complications.

Grade	Definition
Grade I	Any deviation from the normal postoperative course without the need for pharmacological treatment or surgical, endoscopic, and radiological interventions. Allowed therapeutic regimens are: drugs as antiemetics, antipyretics, analgesics, diuretics, electrolytes, and physiotherapy. This grade also includes wound infections opened at the bedside.
Grade II	Requiring pharmacological treatment with drugs other than such allowed for grade I complications. Blood transfusions and total parenteral nutrition are also included.
Grade III	Requiring surgical, endoscopic or radiological intervention
Grade IIIa	Intervention not under general anesthesia
Grade IIIb	Intervention under general anesthesia
Grade IV	Life-threatening complication (including CNS complications)^a^ requiring IC/ICU management
Grade IVa	Single organ dysfunction (including dialysis)
Grade IVb	Multi-organ dysfunction
Grade V	Death of a patient
Suffix ‘d’	lf the patient suffers from a complication at the time of discharge, the suffix „d" (for „disability") is added to the respective grade of complication. This label indicates the need for a follow up to fully evaluate the complication.

CNS: central nervous system; IC: intermediate care; ICU: intensive care unit.

a Brain hemorrhage, ischemic stroke, subarachnoid bleeding, but excluding transient ischemic attacks.

### 2.4 Statistical analysis

Continuous data are reported as median with interquartile range (IQR). Dichotomous variables are shown as frequency (%). Continuous variables were compared using the Wilcoxon rank sum test. Ordinal variables were compared by the Mann Whitney *U* test. Prevalences were calculated as the proportion of appearance divided by the total number in the study population, expressed as a percentage. All statistical analyses were carried out using STATA version 16.1 (StataCorp LLC, Texas, USA). P-values <0.05 were considered as statistically significant.

## 3 Results

### 3.1 Patient cohort

In the study period, according to our patient registry 1065 patients underwent craniotomy for cerebrovascular surgery. SSI occurred in 34 (2.1 %, n = 19 males) cases within 90 days. Median age of the patients with SSI was 53 years (IQR 45–60). The most frequent index surgery was elective clipping of an unruptured intracranial aneurysm (16 cases, 47 %), followed by 7 emergency evacuations of an intracranial hemorrhage together with AVM or cavernoma resection (21 %). Frequencies and patients’ characteristics are listed in Table 2.

**Table 2:** Demographic and baseline clinical data of the whole surgical site infection cohort.

	Whole cohort (n = 34)
Age, median (IQR)	53 (45–61)
Sex, women	15 (44.1)
**Indication of index surgery**	
AVM ruptured	2 (5.88)
AVM unruptured	1 (2.94)
Aneurysm ruptured	4 (11.8)
Aneurysm unruptured	16 (47.0)
Stenosis/ischemia	6 (17.6)
Cavernoma	4 (11.7)
Neurovascular conflict	1 (2.94)
**Type of index surgery**	
AVM or Cavernoma resection	7 (20.6)
Aneurysm clipping	17 (50.0)
EC-IC bypass	4 (11.8)
Decompressive hemicraniectomy	5 (14.7)
Microvascular decompression	1 (2.94)
**Type of surgery for infection**	
Wound revision local anesthesia	2 (5.88)
Wound revision gen. anesthesia Removal of bone flap	32 (94.1) 5 (14.7)
**Revision surgery in emergency department**	
Yes	21 (61.7)
No	13 (38.2)
Interval index surgery to infection, median days (IQR)	27.5 (19–44)
**Modified Rankin scale at admission**	
1	18 (52.9)
2	4 (11.8)
3	3 (8.82)
4	4 (11.8)
5	4 (11.8)
**Modified Rankin scale at discharge**	
0	7 (20.6)
1	13 (38.2)
2	3 (8.82)
3	3 (8.82)
4	3 (8.82)
5	2 (5.88)
6	2 (5.88)
**NIHSS at admission**	
Median (IQR)	0 (0–1)
Mean (SD)	2.1 (4.3)
**NIHSS at discharge**	
Median (IQR)	0 (0–1)
Mean (SD)	1.5 (3.6)
**Use of dexamethasone**	
Preoperatively	1 (2.94)
Postoperatively	0 (0.00)
Immunosuppressed	2 (5.88)
**Antibiotics**	
Preoperatively	2 (5.88)
Postoperatively	32 (94.1)
**Clavien-Dindo Grade**	
Intervention under regional/local anesthesia	2 (5.88)
Intervention under general anesthesia	32 (94.1)

**Fig. 3:**
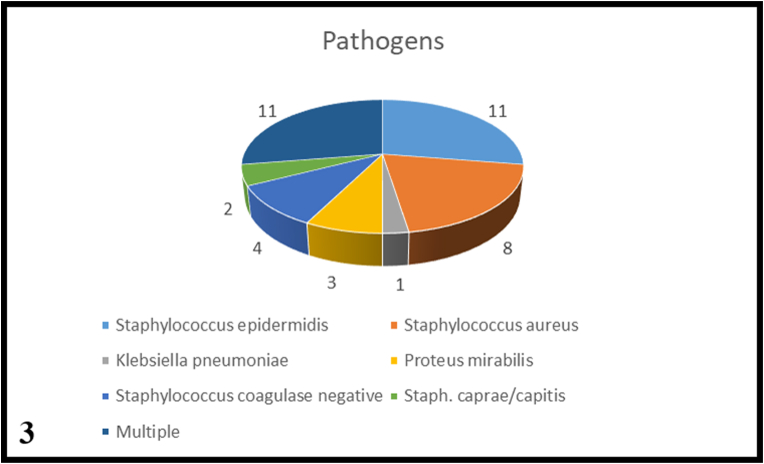
Visual summary of the representation of the pathogens in the cohort.

The median time from the index surgery to surgical wound revision was 27.5 days (IQR 19–44), ranging between 11 and 84 days. Most of the revision procedures (32 cases, 94 %) were performed under general anesthesia and therefore rated as CDG 3b. Only 2 revision procedures (6 %) were performed under local anesthesia. 21 revision surgeries for infection (62 %) took place in the emergency department, whereas the other 13 (38 %) were performed in one of our dedicated neurosurgical operating rooms. The median length of hospital stay after revision surgery was 6.5 days (IQR 3–13). The median mRS at hospital admission and discharge were respectively 1 (IQR 1–3.5) and 1 (IQR 1–3), whereas the medial NIHSS at the same time points were both 0 (IQR 0–1) and a range of 0–16. Between admission for revision surgery and discharge there was no evidence for improvement in the mRS Scale nor in NIHSS (p = 0.259 and p = 0.512, respectively, Wilcoxon rank sum test). Due to the small cohort no further analysis was performed. The microbiological analyses of the intraoperative samples of biological material yielded following results: Staphylococcus epidermidis represented the most common causative microorganism (11 cases, 32 %), followed by Staphylococcus aureus (8 cases, 24 %). Fig. 3 provides a visual summary of the pathogens.

### 3.2 Determination of the relation between infection rate and location of the index surgery

The location of the index surgery could be retrospectively determined in 1053 cases (99.6 %). Location was either in the Emergency Department (ED; n = 319, 30.3 %) or one of our dedicated neurosurgical operating rooms (n = 734, 69.7 %). Three index surgeries (8.8 %) leading to SSI took place in the ED and 30 index surgeries (88.2 %) in our neurosurgical operating rooms. In one patient with SSI the location of the index surgery could not be retrieved. No statistically significant difference of occurrence of SSI depending on OR location was determined (p value 1.0, Wilcoxon rank sum test).

## 4 Discussion

The present study shows an incidence of 2.1 % SSI in patients undergoing craniotomy for cerebrovascular pathologies, with Staphylococcus epidermidis and Staphylococcus aureus being the most common pathogens. Most revisions were performed under general anesthesia and required a prolonged hospital stay. A higher number of SSIs occurred after index surgeries in the ED.

Surgical site infections (SSIs) represent a relevant cause of morbidity in neurosurgical departments. Potential consequences of SSI include often readmission for further therapy and treatment, requiring higher expenses for the health care system (Clavien et al., 2009). Reported rates of SSI after cranial surgery are very limited and show high variability from 0.5 up to 18.8 % (Magni et al., 2024; McClelland, 2008; Lee et al., 2024). To date, there are very few reports on the impact of specific risk factors of SSI in craniotomies, notably featured by high technical complexity and potentially longer intraoperative time (McClelland, 2008; Stienen et al., 2019; Lee et al., 2024).

Our group reported previously an overview on SSI in an unselected series of patients undergoing neurosurgical interventions with a lower overall rate of 2 % than otherwise reported and showed that vascular surgery reached higher rates (Stienen et al., 2019). The rate of surgical site infections (SSIs) in intracranial cerebrovascular procedures may differ from other types of neurosurgical or surgical cases for several reasons. One possible factor is the longer duration of these operations, which is commonly associated with increased infection risk. However, in our own cohort (2013–2024, unpublished data), we did not observe a significant link between surgical time and SSI, making this explanation less convincing in our context. A more likely contributor is the routine use of implants, such as clips, coils, or dural substitutes, which can introduce foreign material into the surgical field and provide surfaces for bacterial adherence or biofilm formation. Another factor worth considering is the changing demographic profile of patients undergoing these procedures. Advances in medical care have made it possible to treat older and more medically complex patients than in the past. As a result, the higher baseline morbidity in this population could also contribute to a different risk profile for SSIs in cerebrovascular surgery compared to other surgical cohorts. Together, these factors may help explain the distinct infection patterns observed in this subset of patients.

The fact that Staphylococcus emerged as the most frequent causative agent of SSIs in our cohort could be due to several overlapping factors. A likely explanation is contamination from the patient's own skin or nasal mucosa, as this microorganism is a common colonizer and may find its way into the surgical field, particularly during longer or more complex procedures (Garrett, 2015). Another possible factor is the local antibiotic resistance profile, which might inadvertently select for this organism despite standard prophylactic measures (Rungelrath and DeLeo, 2021). The type of surgeries performed, often involving vascular reconstruction, the use of implants, and extended operating times, could also create conditions more favorable to infection with S. aureus. In addition, postoperative ICU, which is not uncommon in this patient population, may increase the risk of colonization or cross-infection. While less likely, it's also worth considering that a subset of these patients may have been immunocompromised, potentially increasing their susceptibility. All of these elements together might help explain the pattern we observed.

The strengths of this study are the presentation of real-life incidence and impact of SSIs for patients undergoing cerebrovascular surgeries with data from a prospective quality registry. The impact of SSIs has a minimal bias, because the treatment of the SSI and outcome assessment were performed in a protocolized way and with scores for which the assessors were certified. The merit of a prospective registry was emphasized by a study from the group of Campbell who reported the inadequacy of retrospective data collection of postoperative adverse events, demonstrating an underestimation of complication rates when compared to prospective data collection (Campbell et al., 2011).

### 4.1 Future perspectives

While the continuous collection of clinical data in a prospective patient registry benefits a more objective reporting and the possibility for analyses with minimal bias, larger studies including only prospectively collected data from different centers would be recommended. Identifying and optimizing risk factors for SSI after craniotomy in cerebrovascular surgery may further improve the perioperative management of these patients.

### 4.2 Limitations

The present analysis has several limitations. First, the study spans an extended period, which may have introduced heterogeneity in the pathologies as well as in the demographic and clinical characteristics of the patient population over time. Second, most patients with SSI were detected and treated prior to the implementation of a standardized departmental infection treatment protocol in 2017. Before its introduction, the assessment and management of SSI were not standardized and were instead based on the discretion of the treating physician. This lack of uniformity may have influenced the detection, documentation, and treatment of SSIs during the earlier phases of the study. Indeed a further limitation is the retrospective analysis of the data, which was, nonetheless, collected in a prospective register.

## 5 Conclusions

The incidence of SSI after craniotomy in our population is 2.1 % with a mean time to wound revision of 27.5 days. Most revisions were performed under general anesthesia and required a prolonged hospital stay. Staphylococcus epidermidis, Staphylococcus aureus represented the most common pathogens. These results can be used as a benchmark and reference for further defining incidence of SSI in cerebrovascular disease and may support strategies to reduce postoperative infections in cerebrovascular surgery.

## Declaration of competing interest

With the present document, as the corresponding authors, I declare personally and on behalf of the other co-authors that **none of the authors have disclosures to make** and **no artificial intelligence has been implemented** in any of the phases of the performance of this study.

## References

[bib1] Anderson D.J., Kaye K.S. (2009). Staphylococcal surgical site infections. Infect. Dis. Clin..

[bib2] Borchardt R.A., Tzizik D. (2018). Update on surgical site infections: the new CDC guidelines. J. Am. Acad. Physician Assistants.

[bib3] Campbell P.G., Malone J., Yadla S. (2011). Comparison of ICD-9–based, retrospective, and prospective assessments of perioperative complications: assessment of accuracy in reporting: clinical article. SPIEL.

[bib4] Cassir N., De La Rosa S., Melot A. (2015). Risk factors for surgical site infections after neurosurgery: a focus on the postoperative period. Am. J. Infect. Control.

[bib5] Cerebrovascular surgery - an overview | ScienceDirect topics. https://www.sciencedirect.com/topics/medicine-and-dentistry/cerebrovascular-surgery.

[bib6] Clavien P.A., Barkun J., De Oliveira M.L. (2009). The clavien-dindo classification of surgical complications: five-year experience. Ann. Surg..

[bib7] Dammann P., Breyer T., Wrede K.H., Stein K.P., Wanke I., Grams A.E., Gizewski E.R., Schlamann M., Forsting M., Sandalcioglu I.E., Sure U. (2014). Treatment of complex neurovascular lesions: an interdisciplinary angio suite approach. Ther Adv Neurol Disord.

[bib8] Dindo D., Demartines N., Clavien P.A. (2004). Classification of surgical complications: a new proposal with evaluation in a cohort of 6336 patients and results of a survey. Ann. Surg..

[bib9] Garrett J.H. (2015). A review of the CDC recommendations for prevention of HAIs in outpatient settings. AORN J..

[bib10] Gross W.L., Sacho R.H. (2021). Intracranial vascular procedures. Anesthesiol. Clin..

[bib12] Korol E., Johnston K., Waser N. (2013). A systematic review of risk factors associated with surgical site infections among surgical patients. Khan AU. PLoS One.

[bib13] Lee K.S., Borbas B., Plaha P., Ashkan K., Jenkinson M.D., Price S.J. (2024). Incidence and risk factors of surgical site infection after cranial surgery for patients with brain tumors: a systematic review and meta-analysis. World Neurosurg..

[bib14] Magni F., Al-Omari A., Vardanyan R., Rad A.A., Honeyman S., Boukas A. (2024). An update on a persisting challenge: a systematic review and meta-analysis of the risk factors for surgical site infection post craniotomy. Am. J. Infect. Control.

[bib15] McClelland S. (2008). Postoperative intracranial neurosurgery infection rates in North America versus Europe: a systematic analysis. Am. J. Infect. Control.

[bib16] Rungelrath V., DeLeo F.R. (2021). Staphylococcus aureus, antibiotic resistance, and the interaction with human neutrophils. Antioxidants Redox Signal..

[bib17] Stienen M.N., Moser N., Krauss P., Regli L., Sarnthein J. (2019). Incidence, depth, and severity of surgical site infections after neurosurgical interventions. Acta Neurochir..

[bib18] Zarb P., Coignard B., Griskeviciene J. (2012). The european centre for disease prevention and control (ECDC) pilot point prevalence survey of healthcare-associated infections and antimicrobial use. Euro Surveill..

